# Roles of Bacterial Symbionts in Transmission of Plant Virus by Hemipteran Vectors

**DOI:** 10.3389/fmicb.2022.805352

**Published:** 2022-01-27

**Authors:** Wei Wu, Hong-Wei Shan, Jun-Min Li, Chuan-Xi Zhang, Jian-Ping Chen, Qianzhuo Mao

**Affiliations:** State Key Laboratory for Managing Biotic and Chemical Threats to the Quality and Safety of Agro-Products, Key Laboratory of Biotechnology in Plant Protection of Ministry of Agriculture and Zhejiang Province, Institute of Plant Virology, Ningbo University, Ningbo, China

**Keywords:** plant virus, insect vector, bacterial symbionts, horizontal transmission, vertical transmission

## Abstract

The majority of plant viruses are transmitted by hemipteran insects. Bacterial symbionts in hemipteran hosts have a significant impact on the host life, physiology and ecology. Recently, the involvement of bacterial symbionts in hemipteran vector-virus and vector-plant interactions has been documented. Thus, the exploitation and manipulation of bacterial symbionts have great potential for plant viral disease control. Herein, we review the studies performed on the impact of symbiotic bacteria on plant virus transmission, including insect-bacterial symbiont associations, the role of these bacterial symbionts in viral acquisition, stability and release during viral circulation in insect bodies, and in viral vertical transmission. Besides, we prospect further studies aimed to understand tripartite interactions of the virus-symbiotic microorganisms-insect vector.

## Introduction

Insect-borne viruses have been associated with significant global challenges in humans, animals, and plants. Most plant viruses depend on insect vectors for their survival and transmission. Insects transmit plant viruses *via* three principal modes, non-persistent, semi-persistent, and persistent. This is based on the length of the period the vector can harbor infectious particles, which ranges from minutes to hours (non-persistent), days (semi-persistent) as well as for a lifetime and even inheritance by insect progeny (persistent). For non-persistent and semi-persistent viruses, viral particles are mainly retained by the vector in the stylet (food canal) or foregut, respectively (Ng and Falk, 2006; Hogenhout et al., 2008). Most persistent plant viruses are transmitted by Hemiptera insects (including aphids, whiteflies, leafhoppers, and planthoppers) (Hogenhout et al., 2008; Gray et al., 2014; Jones and Naidu, 2019). During insect sucking, persistent viruses are taken up together with the plant sap. Subsequently, they infect gut epithelial cells, are released into the hemocoel, invade hemocytes, the salivary glands, and other tissues/organs, including nervous systems and reproductive systems (Hogenhout et al., 2008; Wei and Li, 2016; Wilson et al., 2020). The vector insects transmit the plant viruses to healthy plant hosts during the course of sucking plant sap (Blanc et al., 2014; Wei and Li, 2016; Wang and Blanc, 2021). Viruses that replicate in insect bodies are referred to as persistent propagative viruses, while those that do not are referred to as persistent circulative viruses (Hogenhout et al., 2008). Often, propagative viruses infect reproductive systems and can be vertically transmitted between generations of insect vectors (Wei and Li, 2016; Wilson et al., 2020).

Viral dissemination in insect bodies is highly associated with vector competency of viral transmissions. Most studies on transmission mechanisms are based on virus-insect protein interactions as well as host and vector manipulations (Wei and Li, 2016; Wang and Blanc, 2021; Xu et al., 2021). As in mammals and other eucaryotes, insects are inhabited by various symbiont microorganisms and the insect health and basic biology are influenced and modulated by them, including nutritional metabolism, reproduction, and pathogen defense (Douglas, 2011; Bennett and Moran, 2013; Newell and Douglas, 2014). Increasing evidence reveals that symbiotic interactions between insects and microorganisms can also affect the transmission of plant viruses by insect vectors (Moran et al., 2005; Werren et al., 2008). Therefore, symbiotic microbes of insects are also involved in tripartite interactions among plant virus, insect vectors and plant host. Through direct and/or indirect mechanisms, such as immune status regulation, modulation of physical barriers on the intestinal epithelium, or the release microbe-derived components/metabolites, symbionts play intricate roles in regulation of insect vector permissiveness to viruses (Cirimotich et al., 2011; Shane et al., 2018; Wu P. et al., 2019; Ma et al., 2021). Therefore, we review recent studies on the significance of symbiotic microorganisms of insect vectors in regulation of plant virus transmission to elucidate on the multilayered virus-vector-microbial symbiont interactions.

## Associations Between Insects and their Bacterial Symbionts

Various insects live in intimate symbiotic associations with microorganisms, which allows insects to adapt to various ecological environments (Moran, 2007; Duron and Hurst, 2013). Insect viability and mobility, which can be influenced by symbiotic organisms, impacts their abilities as viral vectors. Microbial symbionts provide essential nutrients for insect hosts and regulate the development, reproduction, metabolism, immunity, protection from antagonists, degradation of toxins, as well as host adaptation to a given environment (Hansen and Moran, 2014; Gao et al., 2020). Based on the degree of interdependence between symbionts and their hosts, they can be divided into obligate (primary) and facultative (secondary) symbionts (Buchner, 1965), between which, there are no clear distinctions. This means that, under special circumstances, facultative symbionts can become obligate (Latorre and Manzano-Marin, 2017).

Many insects feed on poor or unbalanced food sources, including sap-sucking hemipteran insects (including aphids, psyllids, planthoppers, leafhoppers, and whiteflies), blood-feeding tsetse flies, cockroaches, weevils, and certain generas of ants (Hansen and Moran, 2014; Rio et al., 2016). Therefore, in most cases, obligate symbionts can supply essential nutrients to these insects (Douglas, 2011). Often, these symbionts exist within specialized, enlarged insect cells referred to as mycetocytes or bacteriocytes, which are integrated into large organs termed mycetomes or bacteriomes. They exhibit a complete dependence on hosts to survive and they cannot be artificially cultured *in vitro*. The bacteriome consists of body fat cells, gut-wall cells, or highly specialized cells that are developmentally determined in the embryonic stage, varying among host groups (Buchner, 1965). In addition, obligate symbionts are strictly vertically transmitted from their mothers to offsprings, usually by infecting oocytes or embryos through various mechanisms (Miura et al., 2003; Gottlieb et al., 2008; Sacchi et al., 2008), or by being encased in “symbiotic shuttles,” that is, symbiont containing capsules that are deposited by females under egg mass (Hosokawa et al., 2007). Consequently, obligate symbionts exhibit hallmarks of long-term co-evolutions with their insect hosts, such as extreme genome reductions (Moran and Bennett, 2014). With regards to facultative symbionts, they may be involved in a broad variety of roles when compared to obligate symbionts, and provide ecological benefits for insect hosts, including adaptation to host plants, body color regulation, heat tolerance, manipulation of host reproduction, pathogenic transmission, defense against natural enemies, and insecticide resistance (Oliver et al., 2003; Tsuchida et al., 2004, 2010; Burke et al., 2010; Hansen and Moran, 2014). Besides, facultative symbionts are distributed in various tissues and cell types of insect vectors and can be transmitted *via* both vertical and horizontal mechanisms (Moran et al., 2008; Moran and Bennett, 2014).

Plant sap-feeding hemipteran insects, such as aphids, whiteflies, leafhoppers and planthoppers, are the most important agricultural pests and serve as vectors for phytopathogenic viruses and bacteria (Hogenhout et al., 2008; Wei and Li, 2016; Wang and Blanc, 2021; Xu et al., 2021). Among them, aphids are polyphagous and transmit over 100 plant viruses (Hogenhout et al., 2008; Chen Y.Z. et al., 2020). Due to the diversity of aphid-associated symbiont communities, they are probably the best model systems for studies on symbiont-host interactions. Nearly all aphids are infected with the obligate endosymbiont, *Buchnera aphidicola* (hereafter referred to as *Buchnera*), which is housed inside bacteriocytes and is strictly vertically transmitted from the mother to offsprings (Dedryver et al., 2010). As an endosymbiont, *Buchnera* provides essential nutrients and vitamins to the aphid host, which cannot be obtained from the diet or from other symbionts (Hansen and Moran, 2011). Through co-inhabitation with *Buchnera*, aphids may also harbor one or several facultative symbionts, which provide ecological benefits to hosts, including defensive behaviors (*Hamiltonella defensa*), reproduction (*Wolbachia*), body color (*Rickettsiella*), host plant fitness (*Regiella insecticola*), heat shock enhancement, parasitoid as well as pathogenic resistance (*Serratia symbiotica*, *H. defensa*, and *R. insecticola*) (Guo et al., 2017).

The whitefly, *Bemisia tabaci*, which can feed on over 600 plants species, including vegetables, fibers, and ornamental crops, is one of the most destructive insect pests. It is a natural vector of persistently transmitted begomoviruses (family *Geminiviridae*) as well as some semi-persistently and non-persistently transmitted plant viruses (Brown and Czosnek, 2002; Wang and Blanc, 2021). *Portiera aleyrodidarum* (hereafter referred to as *Portiera*), the obligate endosymbiont for all whitefly species, is localized in the bacteriome, which comprises several bacteriocytes. It provides essential amino acids, carotenoids, and other metabolites that are scanty in a phloem sap diet to its insect host (Thao and Baumann, 2004; Sloan and Moran, 2012). In whiteflies, *Portiera* is transmitted to progenies through a unique mechanism in which intact bacteriocytes migrate to the ovaries and enter eggs (Luan et al., 2016). In addition to *Portiera*, whitefly species are associated with seven facultative endosymbionts; *Arsenophonus*, *Cardinium*, *Fritschea*, *Hamiltonella*, *Hemipteriphilus*, *Rickettsia*, and *Wolbachia* (Zchori-Fein and Brown, 2002; Nirgianaki et al., 2003; Weeks et al., 2003; Everett et al., 2005; Gottlieb et al., 2006; Bing et al., 2013).

Symbioses in *Auchenorrhyncha* (*Hemiptera*: suborder), such as leafhoppers, treehoppers, cicadas, planthoppers, and spittlebugs, are ancient and complicated. The obligate symbiont, *Sulcia muelleri* (hereafter referred to as *Sulcia*), and one or two co-obligate betaproteobacterial symbionts are associated with many hosts (Buchner, 1965; Bennett and Moran, 2013). Normally, *Sulcia* synthesizes a set of seven or eight essential amino acids, while a co-obligate betaproteobacterial symbiont synthesizes the two or three remaining essential amino acids (Bennett and Moran, 2013). *Sulcia* and the co-obligate betaproteobacterial symbionts are confined to bacteriomes located in host abdomens (Buchner, 1965). In female adults, simultaneously, *Sulcia* and the co-obligate betaproteobacterial symbiont enter same follicular epithelial cells surrounding posterior poles of oocytes and accumulate in oocytes to form a characteristic “symbiont ball” (Pyka-Fosciak and Szklarzewicz, 2003; Michalik et al., 2013; Szklarzewicz et al., 2016). In the leafhopper, some co-obligate betaproteobacterial symbionts are able to live inside cytoplasms of *Sulcia* to ensure simultaneous transovarial transmissions in generations of insects (Michalik et al., 2013, 2014; Kobiałka et al., 2016).

Due to the loss of co-obligate betaproteobacterial symbionts in some *Auchenorrhyncha* species, they have been replaced by novel co-symbionts, such as gammaproteobacteria or yeast-like symbionts (Noda, 1977; Szklarzewicz et al., 2020). For instance, the obligate symbiont, *Sulcia*, in planthoppers was lost and replaced by the yeast-like symbiont (YLS) to provide essential amino acids (Sasaki et al., 1996). However, YLS genomes do not have complete vitamin B synthetic genes, therefore, the facultative symbiont, *Wolbachia*, is required for vitamin B synthesis in the host (Ju et al., 2020). In insects, *Wolbachia* is the most abundant endosymbiont and is best known for altering host reproductive biology to enhance the spread of infections across generations (Stevens et al., 2001).

## Effects of Bacterial Symbionts on Plant Virus Circulation in Insect Bodies

**FIGURE 1:**
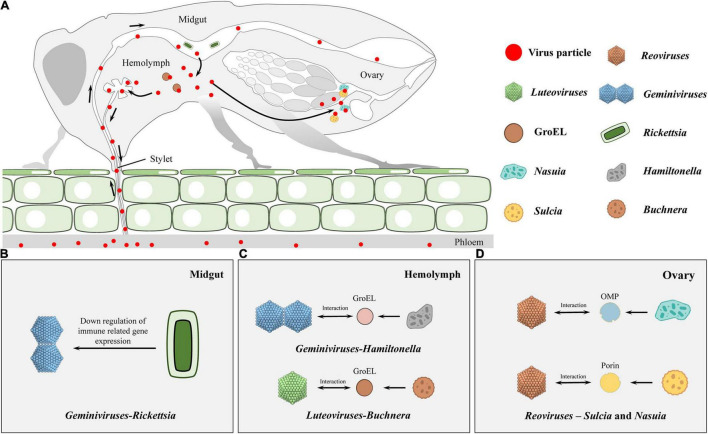
Schematic diagram on infection routes of persistent plant viruses in insect vector. **(A)** During feeding, viral particles are sucked up together with plant sap proteins and ingested into the gut lumen by the insect vectors. Subsequently, the ingested virions initially infect the intestinal epithelium and transfer across the midgut cellular barriers to the hemolymph. Finally, virions enter into salivary glands to be horizontally transmitted to healthy plants or transfer into the reproductive system, thus being vertically transmitted to offspring. **(B,C)** In horizontal transmission, Rickettsia in the midgut lumen can benefit geminivirus acquisition, retention, and transmission in the insect **(B)**. Here, it should be noticed that GroEL-begomovirus/-luteovirus interaction is essential for the stability of the virus in hemolymph, otherwise the insect immune system may recognize and degrade these foreign invaders **(C)**. **(D)** During the process of vertical transmission, Reovirus virions migrate to the ovaries and enter the eggs by hitchhiking on the envelopes of *Sulcia* and *Nasuia*, and they benefit from the vertical transmission routes taken by the two obligate bacterial symbiont partners in females.

In persistent transmissions, plant viruses move through insect bodies and are horizontally transmitted from infected plant hosts to healthy hosts. Suspended viral particles in plant saps are usually taken up by insects through ingestion, they circulate in the gut, the hemolymph and salivary glands, and are finally released into saliva for transmission (Figure 1A; Hogenhout et al., 2008; Wei and Li, 2016; Wang and Blanc, 2021). Within insects, viruses encounter multiple physical and immune barriers, and previous studies often interpret these mechanisms in terms of protein interactions (Jia et al., 2018; Wilson et al., 2020). In fact, symbiotic bacteria influence multiple insect physiologies, such as nutrition, metabolism, reproduction as well as immunity, and can maintain homeostasis within insects through various immune mechanisms (Hansen and Moran, 2014; Gao et al., 2020). Moreover, increasing evidence has revealed that these endosymbionts play intricate roles in modulation of vector susceptibility to viruses and their transmission through direct or indirect mechanisms. Therefore, we elucidated on the roles of symbionts during the cycle of plant viruses in insect bodies.

### Bacterial Symbionts Regulating Viral Acquisition by Insect Vectors

When insect vectors feed on virus-infected plants, the intestinal tract is the first viral-entry site of persistent viruses, therefore, it is the principal determinant for viral transmission by insects (Hogenhout et al., 2008; Wei and Li, 2016; Wilson et al., 2020). Gut commensal microbiome may regulate host defenses against viral infections of gut epithelial cells (Yin et al., 2020), however, the relationship between intestinal symbionts and plant viruses during viral acquisition by insect vectors has not been fully established.

Various studies have reported the involvement of endosymbionts in acquisitions of plant viruses. In the *B. tabaci* whitefly, nearly all facultative symbionts are co-localized with obligate endosymbionts inside bacteriocytes, ensuring vertical transition (Zchori-Fein and Brown, 2002; Nirgianaki et al., 2003; Weeks et al., 2003; Thao and Baumann, 2004; Everett et al., 2005; Gottlieb et al., 2006; Sloan and Moran, 2012; Bing et al., 2013). On the contrary, *Rickettsia* exists outside bacteriocytes, infects all insect organs, and replicates to high levels in the gut, hemolymph, and salivary glands (Gottlieb et al., 2008; Brumin et al., 2012). Kliot et al. (2014) established two isofemale whitefly strains from an inbred B biotype strain (∼300 generations): *Rickettsia* infected (Rick+) and the *Rickettsia* non-infected (Rick–). They showed that *Rickettsia* infections enhance tomato yellow leaf curl virus (TYLCV) acquisition, retention, and transmission by insects (Table 1). The acquisition of TYLCV massively down-regulated immune system gene expressions in the Rick+ population, while in the Rick– population, the virus massively activated immune-related gene expressions (Figure 1B; Kliot et al., 2019). Lei et al. (2021) documented that interactions between the rickettsial secretory protein, BtR242, and protein coat of the cotton leaf curl Multan virus (CLCuMuV), was beneficial in CLCuMuV transmission by whiteflies (Table 1). Moreover, vitellogenin (Vg) levels in the Rick+ population was more than twofold higher than that of the Rick– population. Biologically, Vg facilitates TYLCV movement across the midgut barrier of its insect vector, *B. tabaci* (He et al., 2021). It has been reported that gut microbiota are not involved in persistent plant virus transmissions by insect vectors, for instance, gut microbiota of thrips larvae did not influence TSWV transmissions (Table 1; De Vries et al., 2012); Wheat dwarf virus (WDV) altered the gut microbiota through a dynamic and reversible manner, while viral transmission was not affected by gut microbiota diversity and abundance in the leafhopper (Table 1; Wang et al., 2019).

**TABLE 1:** Summary of the interactions of insect endosymbionts with plant viruses demonstrated by *in vitro* or *in vivo* experiments.

Insect	Endosymbiont	Endosymbiont product	Virus	Effect on transmission	References
*Myzus persicae*	Unspecified	Undetermined	Potato leaf roll virus	Endosymbiotic bacteria play a crucial role in determining the persistent nature of PLRV in the aphid hemolymph.	Van den Heuvel et al., 1994
*M. persicae*	Unspecified	Symbionine (GroEL homolog)	Potato leaf roll virus	The absence of GroEL homolog in the hemolymph of aphids after treated with antibiotics leads to virus degradation and concomitant loss of infectivity.	Hogenhout et al., 1996
*R. padi Sitobion avenae*	Unspecified	SymL (GroEL homolog)	Barley yellow dwarf virus	Endosymbiotic SymL interacted with BYDV RTD.	Filichkin et al., 1997
*M. persicae Acyrthosiphon pisum Rhopalosiphum padi*	*Buchnera*	GroEL	Beet western yellows virus, Beet mild yellowing virus, Potato leaf roll virus, Cucurbit aphid-borne yellows virus, Bean leafroll virus Soybean dwarf virus Pea enation mosaic virus	The N-terminal region of the luteovirus RTD determines virus binding to *Buchnera* GroEL and is essential for the stability of virions in hemolymph.	van den Heuvel et al., 1997
*M. persicae*	*Buchnera*	GroEL	Potato leaf roll virus	The interaction site between PLRV and *Buchnera* GroEL is located in the equatorial domain.	Hogenhout et al., 1998
*Bemisia tabaciis*	Unspecified	GroEL homolog	Tomato yellow leaf curl virus	No TYLCV viral DNA was detected in the hemolymph of whiteflies fed with anti-GroEL antibodies prior to virus acquisition.	Morin et al., 1999
*A. pisum*	Undetermined	Undetermined	Pea enation mosaic virus	The RTD is not necessary for stability of PEMV in the aphid hemolymph.	Liu et al., 2009
*A. pisum R. padi*	*Buchnera*	GroEL	Barley yellow dwarf virus	GroEL was detected in bacteriocyte, but not in the aphid hemolymph, fat body or gut.	Bouvaine et al., 2011
*Schizaphis graminum*	*Buchnera*	Undetermined	Cereal yellow dwarf virus	The genotype of *Buchnera* correlates with the ability to efficiently transmit CYDV by aphid.	Cilia et al., 2011
*B. tabaciis*	*Hamiltonella*	GroEL	Tomato yellow leaf curl virus	The GroEL protein produced by *Hamiltonella* facilitates TYLCV transmission.	Gottlieb et al., 2010
*Bemisia tabaci*	*Arsenophonus*	GroEL	Cotton leaf curl virus	*Arsenophonus* involve in the transmission of CLCuV in whitefly.	Rana et al., 2012
*Frankliniella occidentalis*	*Erwinia sp.*	Unspecified	Tomato spotted wilt virus	TSWV transmission is not affected by the number of the symbiotic bacteria Erwinia sp. present in the gut of thrips larvae.	De Vries et al., 2012
*B. tabaciis*	*Hamiltonella*	Unspecified	Tomato yellow leaf curl virus	*Hamiltonella* is closely associated with the acquisition, retention and transmission efficiency of TYLCV by the whitefly.	Su et al., 2013
*Bemisia tabaci*	*Rickettsia*	Undetermined	Tomato yellow leaf curl virus	*Rickettsia* increases TYLCV transmission efficacy by infecting the midgut.	Kliot et al., 2014
*Nephotettix cincticeps*	*Sulcia*	Out membrane protein	Rice dwarf virus	*Sulcia* supporting RDV transfer to the next generation.	Jia et al., 2017
*B. tabaciis*	*Hamiltonella*	Unspecified	Cowpea mild mottle virus, Bean golden mosaic virus, Tomato chlorosis virus	*Hamiltonella* increased the transmission efficiency of begomovirus by the whitefly.	Bello et al., 2019
*Bemisia tabaci*	*Rickettsia*	Undetermined	Tomato yellow leaf curl virus	*Rickettsia* down-regulates of whitefly immunity genes to increase the ability of whitefly to acquire, retain and transmit TYLCV.	Kliot et al., 2019
*Nephotettix cincticeps*	*Nasuia*	Prion	Rice dwarf virus	*Nasuia* supporting RDV transfer to the next generation.	Wu W. et al., 2019
*Psammotettix alienus*	Unspecified	Unspecified	Wheat dwarf virus	WDV changes the gut microbiota by a dynamic and reversible manner, while the virus transmission was not affected by the diversity and abundance of gut microbiota in leafhopper.	Wang et al., 2019
*Nilaparvata lugens Laodelphax striatellus*	*Wolbachia*	Unspecified	Rice ragged stunt virus	The Wolbachia strain *w*Stri (Isolated from the small brown planthopper, *Laodelphax striatellus*) has been stably introduced into brown planthopper, *Nilaparvata lugens* and shown to inhibited infection and transmission of Rice ragged stunt virus (RRSV) and mitigated virus-induced symptoms in rice plants.	Gong et al., 2020
*Bemisia tabaci*	*Rickettsia*	Unspecified	Cotton leaf curl multan virus	*Rickettsia* enhances the transmission efficiency of the CLCuMuV by whitefly.	Lei et al., 2021

Given that most gut symbiotic microorganisms are obtained from food, the diversity of intestinal symbiotic microorganisms in sap-feeding insects is significantly low due to low microbial contents in plant sap (Jing et al., 2014). This is a major limitation of studies on direct interactions between intestinal microbes and plant viruses in insect gut cell infections. In arboviruses, gut microbiota regulate viral infections in gut epithelial cells by modulating gut immune responses, altering the physical status, or by directly utilizing microbiota-derived products (Wu P. et al., 2019; Ma et al., 2021). For instance, *Proteus sp*., which is a mosquito intestinal symbiotic bacteria, suppresses dengue virus (DENV) infection by enhancing the expression levels of antimicrobial peptides in gut epithelial cells (Ramirez et al., 2012), while another gut commensal, *Serratia marcescens*, facilitates arboviral infections by secreting the *Sm*Enhancin protein, which digests gut membrane-bound mucins, thereby enhancing viral dissemination in mosquitoes (Wu P. et al., 2019). The gut commensal, *Chromobacterium* sp., secretes an aminopeptidase that is able to degrade the DENV envelope protein, preventing their attachment and infection of host cells (Saraiva et al., 2018).

### Bacterial Symbionts Contributing to the Stability of Plant Viruses in Vector Hemolymph

The insect hemolymph, which is critical in persistent viral transmissions by insect vectors, can be used as a bridge to the salivary glands (Liu et al., 2015; Chen X. et al., 2020). Besides, it is well-defended by the immune system, which can effectively remove microorganisms (such as bacteria, fungi, and viruses) (Lavine and Strand, 2002; Blow and Douglas, 2019). Even though obligate symbionts have long-term coevolutions with their insect hosts, they can only exist within specialized cells (mycetocytes or bacteriocytes) to escape the immune system (Gross et al., 2009). After viral particles are released from the midgut to the hemolymph, they can be recognized, targeted and cleared by the host immune system. Therefore, survival of virions within the hemolymph is vital for systemic dissemination of persistent viruses before entry into salivary glands (Liu et al., 2015; Chen X. et al., 2020).

Van den Heuvel et al. (1994) reported that chaperone proteins of endosymbionts may be involved in maintenance of plant viruses in the hemolymph. They used a virus overlay assay to search for proteins that interacted with the Potato leafroll virus (PLRV) in the green peach aphid *Myzus persicae* Sulzer (Homoptera: Aphididae) and revealed that PLRV binds the symbionin expressed by the obligate endosymbiont of *M. persicae, Buchnera* (Table 1). Administration of antibiotics to aphids was associated with the absence of symbionin in the hemolymph, which led to viral degradation, loss of infection, and a 70% reduction in transmission efficiencies (Table 1; Van den Heuvel et al., 1994; Hogenhout et al., 1996). Symbionin, which has a high homology with the *Escherichia coli* heat shock protein, GroEL, is a chaperone that is highly conserved from bacteria to all multicellular life forms and is generally composed of 14–18 subunits arranged in two rings. It enhances proper folding of complex, multidomain proteins and is involved in the maintenance of protein homeostasis (Piana and Shaw, 2018). Studies involving aphids and luteoviruses revealed that all luteoviruses bind GroEL proteins (with different affinities), while purified luteovirus particles contain a major 22-kDa coat protein (CP) and less amounts of an approximately 54-kDa readthrough protein (RTD), expressed by translational readthrough of the CP into the adjacent open reading frame. Beet western yellow luteovirus (BWYV) mutants, lacking RTD, did not bind *Buchnera* GroEL (Table 1). However, mutants with deletions only at the C-terminal of RTD bound as efficiently as wild-type BWYV. These findings imply that the conserved N-terminal of RTD is part of the luteovirus capsid that is required for binding of GroEL to virions (Figure 1C; Filichkin et al., 1997; van den Heuvel et al., 1997). *In vitro* interaction studies showed that PLRV binds the equatorial domain of *Buchnera* GroEL (Hogenhout et al., 1998).

The involvement of GroEL in luteovirus transmission has not been conclusively determined. Liu et al. reported that Pea enation mosaic virus (PEMV) mutants devoid of the readthrough domain (RTD) exhibited the same stability as wild-type viruses in aphid hemolymphs (Table 1). This indicates that RTD is not necessary for PEMV stability in aphid hemolymph (Liu et al., 2009). In addition, through immunoblotting and immunocytochemistry, Bouvaine et al. (2011) reported that GroEL was restricted to the bacteriocyte and was never detected in aphid hemolymph, fat body or gut (Table 1). Therefore, *Buchnera* GroEL was not available to interact with luteoviruses *in vivo* (Bouvaine et al., 2011). Furthermore, Bouvaine et al. (2011) considered that specific detection of GroEL in the hemolymph is depended on the method used for aphid dissection. In the hemolymph, GroEL can be detected if cornicle amputation is used for hemolymph collection, but it is never detected when leg amputation is performed to obtain hemolymph (Bouvaine et al., 2011). Through proteomics and genetics experiments, Cilia et al. (2011) evaluated the linkage between *Buchnera* genotypes and the ability to transmit Cereal yellow dwarf virus-RPV (CYDV-RPV) (Table 1). They proved that one *Buchnera* genotype is required for viral transmission (Cilia et al., 2011). Taken together, these studies do not prove or disapprove that *Buchnera* GroEL contributes to the stability of luteoviruses in aphid hemolymph. Therefore, the mechanism through which GroEL protects the virus during hemolymph translocation may not be the same for all luteoviruses.

Similar to aphid-transmitted luteoviruses, geminivirus is transmitted in a circulative manner by whiteflies. A GroEL homolog produced by *Hamiltonella*, a facultative endosymbiont of the whitefly, showed 80% homology with that from different aphid species and GroEL from *E. coli*. Feeding the anti-*Buchnera* GroEL antiserum to *B. tabaci* whitefly before viral acquisition inhibited TYLCV transmission by more than 80% (Figure 1C, Table 1; Morin et al., 1999). In addition, the GroEL expressed by *Hamiltonella*, a facultative endosymbiont of *B. tabaci*, exhibited 80% homology with *Buchnera* GroEL, and specifically interacted with the TYLCY CP (Gottlieb et al., 2010). Su et al. (2013) cultured two isofemale strains [*Hamiltonella* infected (H+) and *Hamiltonella* non-infected (H–)] from a same genetic background insect strain *via* antibiotic treatment and introgression. Through further studies, they found that whiteflies harboring *Hamiltonella* were suitable for TYLCV acquisition, retention, and spread (Table 1). Moreover, the abundance of *Hamiltonella* on whiteflies has been associated with the transmission efficiency of non-circulative viruses (Table 1; Bello et al., 2019). The GroEL protein of *Arsenophonus* (another facultative symbiotic bacterium of whitefly) was also found to interact with the Cotton leaf curl virus *in vitro* and *in vivo* (Table 1; Rana et al., 2012). In summary, GroEL-plant virus interactions are not restricted to begomoviruses, and they may be applicable to all whitefly transmitted plant viruses.

### Bacterial Symbionts Influence Plant Virus Infections

Escape from insect salivary glands and injection into plant cells with saliva during insect feeding are crucial steps in persistent viral transmission (Hogenhout et al., 2008; Wei and Li, 2016; Wang and Blanc, 2021). Insect salivary glands consist of principal salivary glands and accessory salivary glands, while salivary gland cells are filled with abundant apical plasma membrane lining cavities, where saliva is stored. Plant viruses escape across membrane barriers through specific virus-insect protein interactions (Wei and Li, 2016; Mao et al., 2017), and it has not been determined whether symbionts play a role. Nevertheless, apart from overcoming salivary gland barriers, the stability and infective abilities of viral particles during salivating into plant cells, as well as insect feeding behaviors are greatly involved in the success of inoculation.

The significance of endosymbiotic GroEL in plant virus transmission has been inconclusively discussed. GroEL can bind viruses *in vivo* as well as *in vitro*, and many plant circulative viruses interact with it to avoid destruction in hemolymphs of their insect vectors, such as luteoviruses and *Buchnera* GroEL in aphids, as discussed above. *Buchnera* GroEL has also been detected in saliva (Table 1; Chaudhary et al., 2014), indicating that it plays a role (such as aiding viral survival in saliva) during plant infections by viruses. Nevertheless, transgenic *Nicotiana benthamiana* plants expressing GroEL exhibited tolerance to TYLCV and cucumber mosaic virus (CMV) that showed interactions with GroEL, but not to grapevine virus A (GAV) or tobacco mosaic virus (TMV) that did not show any interactions (Table 1; Edelbaum et al., 2009). The binding to GroEL by viruses in plant sap inhibits them from infecting plants. Besides, the delivery of GroEL into tomato and *Arabidopsis* plants can trigger plant defense systems, pattern-triggered immunity (PTI), which is detrimental to aphid fecundity (Chaudhary et al., 2014). Therefore, expressions of GroEL in plants has the potential for controlling some viral diseases and their insect vectors. These studies suggest that bacterial symbionts in insects may impact plant infection by exerting indirect effects through the modulation of plant defense pathways.

In their study on CMV transmission by the green peach aphid, *M. persicae*, Shi et al. (2021) reported that CMV can switch the insect vector’s feeding preference by affecting the abundance of the obligate endosymbiont, *B. aphidicola*. However, as a non-persistent virus that is transmitted by aphids, through its CP, CMV only binds the stylet of insects. It has been documented that *B. aphidicola*, an aphid endosymbiont, influences herbivore behaviors by modulating plant volatile profiles, implying that CMV infections reduce the abundance of *B. aphidicola* in aphids, which has been associated with a preference shift in aphids from infected to healthy plants (Shi et al., 2021). It is revealed that the symbiotic bacteria affect feeding tropisms and feeding behaviors of insects and influences plant infections by viruses (Frago et al., 2012; Simon et al., 2017; Khan et al., 2019; Noman et al., 2020).

## The Roles of Bacterial Symbionts in Vertical Transmissions of Plant Viruses by Insects

Apart from horizontal viral transmissions between host plants and insect vectors, some persistent viruses can be vertically transmitted from virus-infected parents to their offsprings through maternal or paternal transmissions (Hogenhout et al., 2008; Wei and Li, 2016; Wang and Blanc, 2021). Vertical viral transmissions between generations of insect vectors can guarantee survival during adverse conditions for horizontal transmission, directly affecting viral ecology and epidemiology.

Maternal transmissions of plant viruses through transovarial passage is the most common mode of vertical transmission. During transovarial passage vertical transmission, the virus must pass through the membrane and tissue barriers of the ovary to infect oocytes (Hogenhout et al., 2008; Wei and Li, 2016; Wang and Blanc, 2021). The reproductive system of female insects is made up of a pair of ovaries, each of which contains several ovarioles, consisting of the terminal filament, germarium, vitellarium, and pedicel. Additionally, oocytes produced by the germarium are linearly arranged within the vitellarium, and surrounded by a layer of follicular cells (Büning, 1994; Szklarzewicz et al., 2007). Vitellogenin (Vg), a female-specific protein, is synthesized in the fat body, secreted into the hemolymph and absorbed by receptor-mediated endocytosis of the growing oocytes, thereby providing essential nutrients for embryonic development (Tufail and Takeda, 2008). Furthermore, the rice stripe virus (RSV) and TYLCV can directly bind Vg and enter oocytes from the germarium and follicular cells by appropriating the Vg transport route (Huo et al., 2014; Wei et al., 2017). Interestingly, Vg is also involved in mediating some facultative symbionts into the oocyte (Herren et al., 2013; Guo et al., 2018).

Obligate symbionts exhibit long-term co-evolutions with host insects, and are internalized into essential insect components, which are specifically manifested by the fact they are kept within specialized, enlarged insect cells, and are strictly vertically transmitted from the mother to offsprings (Buchner, 1965; Miura et al., 2003; Gottlieb et al., 2008; Sacchi et al., 2008). Obligate symbiotic bacteria have their own unique mechanisms through which they can gain entry into the ovary (from the posterior pole of the oocyte), in contrast to most plant viruses and facultative symbionts (Szklarzewicz and Michalik, 2017). Among the obligate symbiotic bacteria, an unusual phenomenon (termed “nested symbiosis”) of symbiotic bacteria within other bacteria is sometimes found (Von Dohlen et al., 2001). For instance, *Sodalis* and *Arsenophonus*, facultative γ-proteobacterium symbionts of *Cicadella viridis* and *Macrosteles laevis* leafhoppers can pass through the envelope of the obligate symbiont *Sulcia* (Michalik et al., 2014; Kobiałka et al., 2016). Consequently, *Sodalis* and *Arsenophonus* can directly enter the cytoplasms of obligate bacteria to ensure their simultaneous transmission through insect generations (Michalik et al., 2014; Kobiałka et al., 2016). In some leafhoppers, Vg is incorporated into obligate symbionts, after which bacterial symbionts are exploited as independent carriers into oocytes (Mao et al., 2020). In conclusion, some insect components and microorganisms in insects can use obligate symbiotic bacteria as transporters and hitchhike the existing pathways to facilitate the entry of bacterial symbionts into insect oocytes.

The rice dwarf virus (RDV), belonging to the genus *Phytoreovirus* in the family Reoviridae, is an icosahedral, double-layered particle. It has a diameter of 70 nm, consists of one minor outer capsid protein P2 and one major outer capsid protein P8 (Omura et al., 1989, 1998). The green rice leafhopper, *Nephotettix cincticeps*, the main vector for RDV, is associated with two types of obligate symbiont bacteria—*Sulcia* and *Nasuia* (Honda et al., 2007; Noda et al., 2012). Besides, during their joint transovarial transmissions to the next insect generation, RDV, *Sulcia*, and *Nasuia* form complex tripartite interactions (Figure 1D; Jia et al., 2017). RDV virions migrate to ovaries and enter eggs by hitchhiking on *Sulcia* and *Nasuia* envelopes, and they simply benefit from vertical transmission routes taken by the two obligate bacterial symbiont partners in female *N. cincticeps*. In addition, RDV exploits its minor outer capsid protein P2 to interact with the BSA domain of OMP in the *Sulcia* envelope, inducing the formation of virus-containing invaginations or membrane-enclosed vesicles (Figure 1D, Table 1; Jia et al., 2017). In the case of *Nasuia*, through its major outer capsid protein P8, RDV interacts with porins in bacterial envelopes, thereby inducing the opening of porin channels for virions to pass through the outer membrane and into the periplasmic space (Figure 1D, Table 1; Wu W. et al., 2019). In this case, long-term coexistence of bacterial symbionts and plant viruses on their pathway into oocytes may result in specific evolutionary outcomes for cross-kingdom interactions between a virus and a bacterium in nature.

## Conclusion

In summary, horizontal and vertical transmission of persistent plant viruses by vector insects involve complex interactions among viruses, vector components, and symbionts. In recent years, symbiotic bacteria that regulate the spread of persistent plant viruses through its insect vector have been extensively studied. For instance, *Rickettsia* has been shown to promote TYLCV acquisition, retention, and transmission through insects (Kliot and Ghanim, 2013; Kliot et al., 2019), GroEL maintains the stability of *Geminivirus* and *Luteovirus* virions in hemolymph (Hogenhout et al., 1998; Morin et al., 1999), *Sulcia* and *Nasuia* facilitate the transfer of RDV to the next generation (Jia et al., 2017; Wu W. et al., 2019). Elsewhere, studies have shown that gut microbiota in leafhopper and thrip do not influence the viral transmission efficiency (De Vries et al., 2012; Wang et al., 2019). Overall, the symbiotic microorganisms of various tissues play important regulatory roles in the transmission of persistent plant viruses through its insect vector. Therefore, bacterial symbionts have great potential for plant viral disease control. Up to now, the most successful strategy for viral disease control by symbiotic microorganisms is the introduction of certain strains of Wolbachia into *A. aegypti* for protecting humans from mosquito-borne diseases. Simlarly, the Wolbachia strain *w*Stri (Isolated from the small brown planthopper, *Laodelphax striatellus*) has been stably introduced into brown planthopper, *Nilaparvata lugens* and shown to inhibited infection and transmission of Rice ragged stunt virus (RRSV) and mitigated virus-induced symptoms in rice plants (Gong et al., 2020).

Although knowledge about the interplay among symbiotic microorganisms, insect vectors, and plant virus has rapidly expanded, there are several issues need further investigation. For instance, intestinal microbiota have been found to modulate immune responses, influence the physical status, or directly utilize microbiota-derived products to promote or inhibit arbovirus infection (Wu P. et al., 2019; Ma et al., 2021). Are there other symbionts involved in the regulation of persistent plant virus transmission through insect vectors? How do symbionts modulate virus infection in the insect vector? There is evidence that insect symbionts play important roles in insect-plant interactions (Frago et al., 2012). Virus-host plant-insect vector interactions mediate the process of virus release from salivary glands to infect host plants. Do symbionts influence virus transmission by regulating the tripartite interactions of the virus-host plant-insect vector? To answer this question, in-depth understanding of the interactions among the virus, symbionts, insect vector, and host plant during persistent plant virus transmission through insect vectors is required. New approaches based on the large-scale high-throughput quantitative omics technologies including genomics, proteomics, metabolomics and transcriptomics will provide new opportunities to unravel the multiple interactions in the process of virus transmission by vector insects. Overall, the insights into insect–symbiotic microorganism–virus interactions may provide novel strategies for viral disease prevention in the future.

## Author Contributions

WW, H-WS, and QM: conceptualization. WW and QM: writing original draft preparation. WW, H-WS, J-ML, C-XZ, J-PC, and QM: writing, review, and editing. All authors have read and agreed to the published version of the manuscript.

## Conflict of Interest

The authors declare that the research was conducted in the absence of any commercial or financial relationships that could be construed as a potential conflict of interest.

## Publisher’s Note

All claims expressed in this article are solely those of the authors and do not necessarily represent those of their affiliated organizations, or those of the publisher, the editors and the reviewers. Any product that may be evaluated in this article, or claim that may be made by its manufacturer, is not guaranteed or endorsed by the publisher.
